# Association of Fetal Growth Retardation with Postnatal Osteoprotegerin Concentrations and Aortic Intima–Media Thickness

**DOI:** 10.3390/diseases14030100

**Published:** 2026-03-08

**Authors:** Ageliki A. Karatza, Eirini Kostopoulou, Sotirios Fouzas, Nikolaos Antonakopoulos, Xenophon Sinopidis, Dimitra Kritikou, Alexandra Efthymiadou, Gabriel Dimitriou, Dionysios Chrysis

**Affiliations:** 1 Department of Paediatrics, University of Patras Medical School, 26504 Patras, Greece; karatza@upatras.gr (A.A.K.); sfouzas@upatras.gr (S.F.); xsinopid@upatras.gr (X.S.); kritikoud@upatras.gr (D.K.); eythymiadou@upatras.gr (A.E.); gdim@upatras.gr (G.D.); dchrysis@upatras.gr (D.C.); 2 Fetomaternal Unit, Department of Obstetrics and Gynaecology, University of Patras Medical School, 26504 Patras, Greece; nantonakop@upatras.gr

**Keywords:** fetal growth retardation (FGR), developmental origins of health and disease (DOHAD), osteoprotegerin, aortic intima–media thickness, cardiovascular disease, infants

## Abstract

Background: Fetal Growth Retardation (FGR) is considered a risk factor for atherosclerosis and coronary artery disease in adulthood. Osteoprotegerin (OPG), a member of the tumor necrosis factor receptor superfamily, is reported to be elevated in atherosclerosis. Objectives: In this case-control study, we investigated whether FGR affects postnatal OPG serum concentrations and the possible association between OPG levels and aortic intima–media thickness (aIMT), an index of preclinical atherosclerosis. Methods: We studied 30 infants with FGR and 30 appropriate for gestational age (AGA) infants matched for gestational age and sex. Quantitative determination of plasma OPG was performed via enzyme immunoassay on the second (DOL2) and fifth (DOL5) day of life. aIMT was measured in the distal abdominal aorta and adjusted for aortic lumen diameter. Results: Infants with FGR had significantly higher OPG levels on both DOL2 and DOL5 as compared to controls (DOL2: 5.4 ± 1.0 pmol/L vs. 4.6 ± 1.0 pmol/L, *p* = 0.002 and DOL5: 5.1 ± 0.8 pmol/L vs. 3.9 ± 0.7 pmol/L, *p* < 0.001). Between DOL2 and DOL5, OPG concentrations did not change significantly in infants with FGR (difference 0.3 ± 0.2 pmol/L, *p* = 0.087) but decreased slightly in controls (difference 0.7 ± 0.3 pmol/L, *p* = 0.003). FGR was also associated with increased aIMT (0.11 ± 0.03 vs. 0.06 ± 0.02, *p* < 0.001). There was a positive correlation between OPG and aIMT on DOL2 (r = 0.494, *p* < 0.001), which became stronger on DOL5 (r = 0.791, *p* < 0.001). Conclusions: We report significantly increased concentrations of OPG in infants with FGR and a positive correlation with aIMT. Follow-up studies with repeat OPG and aIMT measurements may be indicated to evaluate whether these findings represent a permanent effect of FGR on the offspring.

## 1. Introduction

Poor fetal growth and small size at birth are followed by increased risk of cardiovascular and metabolic complications later in life. This suggests that these disorders originate in part through unbalanced nutrition in utero and during infancy [1,2,3]. The concept that early life conditions can significantly impact adult health is known as the “fetal origins of disease” or the “developmental origins of health and disease (DOHaD)” and more precisely suggests that the first 1000 days from conception until age 24 months is a critical life stage that can affect long-term health [4]. Fetal growth retardation (FGR) refers to a pathological retardation of intrauterine growth velocity [1,2,3,5,6,7] due to placenta failure to provide an adequate supply of oxygen and nutrients to the developing fetus. It represents a common pregnancy complication affecting 10–15% of all pregnancies worldwide, and may be caused by maternal, fetal, or, most commonly, placental deficiency. It can be associated with increased risk of early complications, such as fetal or neonatal demise, perinatal asphyxia, meconium aspiration syndrome, hypoglycemia, polycythemia, hyperviscosity, non-physiological hyperbilirubinemia, sepsis, hypocalcemia, poor thermoregulation, and immunological incompetence [5,6,7].

Currently, there is no uniform definition for FGR in the literature [1,8]. The first final international consensus suggests that FGR should be defined as birth weight below the third percentile on population-based or customized growth charts (86%) or the presence of at least 3 out of 5 of the following: birth weight < 10th percentile on population-based (78%) or customized growth charts (94%), head circumference < 10th percentile (82%), length < 10th percentile (82%), prenatal diagnosis of FGR (88%), and maternal pregnancy complications (i.e., hypertension or preeclampsia) (78%) [9]. Often, distinguishing between a naturally small fetus and one with pathologically restricted growth remains a challenge. Constitutionally small fetuses are in accordance with their genetic growth potential; therefore, although they are small for gestational age, they are not growth-restricted and not at risk of the severe long-term consequences of FGR [1,6,7].

The identification of novel risk factors and biomarkers of early atherosclerosis may enhance the understanding of the pathophysiological processes implicated in atherosclerosis and the therapeutic approach of potential cardiovascular complications. One of the novel biomarkers currently under investigation is osteoprotegerin (OPG), a TNF-like protein secreted by osteoblasts. The primary biological action of OPG is on osteoclast differentiation. Specifically, receptor activator of nuclear factor-kappa B ligand (RANKL) is a ligand that binds to receptor activator of nuclear factor-kappa B (RANK) on osteoclasts, dendritic cells, T cells and other cells. RANKL signaling promotes osteoclast differentiation and activation that leads to bone resorption. OPG acts as a decoy receptor for RANKL, preventing its interaction with RANK, thus inhibiting osteoclastogenesis and bone resorption [10]. Therefore, the RANK–RANKL–OPG system is critical for bone metabolism, but also for immune function and cancer [10].

A positive association between OPG levels and cardiovascular morbidity and mortality has been reported by several studies, thus rendering OPG a potential marker of cardiovascular risk [11]. Significantly higher OPG plasma concentrations have been confirmed in patients with coronary artery disease, unstable angina, and acute myocardial infarction [11]. In addition, the RANK–RANKL–OPG signalling pathway is thought to be the primary regulator of the progression of blood vessel wall calcification, presumably through a complex pathway that involves alterations in calcium deposition and structural protein synthesis in the arterial wall. In addition, increased OPG expression has been shown to affect platelet adhesion, causing a predisposition to the formation of atherosclerotic plaque [12]. There is mounting evidence that OPG also contributes to the development of atherosclerosis and cardiovascular diseases by intensifying the consequences of inflammation and several traditional risk factors, such as hyperlipidemia, diabetes mellitus, and hypertension [13]. It has been proposed that OPG contributes to cardiovascular disease by causing endothelial dysfunction through blocking RANKL, which activates protective pathways, such as nitric oxide synthase, in endothelial cells [14]. Interestingly, OPG concentrations in the aortic wall are 500-fold higher than in plasma, approaching those in bone. There is minimal information regarding serial OPG and RANKL concentrations in the neonatal and infantile period [15,16].

Currently, there are adequate available data concerning non-invasively measurable alterations in the developing vascular tree of infants, children, and adolescents [4,17,18,19]. Early lesions can be detected and quantified using specific ultrasound indices, such as aortic intima–media thickness (aIMT) and carotid intima–media thickness (cIMT). Abdominal aIMT is a more sensitive marker of subclinical atherosclerosis compared to cIMT in the pediatric population, as early lesions, including fatty streaks, are more likely to develop in the dorsal wall of abdominal aorta [4,17]. Therefore, a progressively increasing number of fetal and neonatal studies have measured aIMT, and several studies have verified increased aIMT in FGR infants [20,21,22,23,24,25,26,27]. Aortic intima-media thickness can be measured reproducibly and non-invasively in infants and young children by external ultrasound [4,17,18].

This prospective case-control study aimed to investigate postnatal OPG concentrations and their association with aIMT in newborns with and without FGR, matched one-to-one for gestational age. The evaluation of a recognized inflammatory marker, such as OPG, with a robust marker of vascular health, such as aIMT, may offer valuable information regarding the presence of subclinical atherosclerosis and potential cardiovascular risk factors in FGR infants since birth, and may contribute to the enhancement of antenatal and postnatal interventions to decrease the burden of premature atherosclerosis in this population.

## 2. Materials and Methods

### 2.1. Study Design and Population

This prospective case-control study was conducted at the University General Hospital of Patras, Greece, a tertiary care referral institution that provides advanced, free, university-level Obstetric and Neonatal services in a region of approximately 700,000 inhabitants. FGR was diagnosed in accordance with the Royal College of Obstetricians and Gynaecologists guidelines [28]. Case subjects were otherwise healthy newborn infants with birth weight < 10th percentile for gestational age and diagnosed FGR, defined as (1) estimated fetal weight or abdominal circumference < 3rd centile or (2) estimated fetal weight or abdominal circumference < 10th centile with abnormal uterine and/or umbilical artery Doppler (pulsatility index > 95th centile and/or absent or reversed end diastolic flow, respectively) [28]. The control group consisted of healthy infants with birth weight appropriate for gestational age (AGA) (10th–90th percentile for gestational age), matched one-to-one with the FGR infants regarding gestational age. Control group growth was assigned using the Fenton third-generation sex-specific growth charts for preterm infants [29]. Gestational age was calculated from the first day of the last menstrual period and by using second-trimester ultrasound estimates. Exclusion criteria for both groups included multiple gestation, congenital malformations or chromosomal abnormalities, congenital heart disease, congenital or neonatal infection, pulmonary hypertension, perinatal asphyxia, tachypnea or respiratory support requirement for more than 12 h, need for volume expansion or administration of inotropes and a hemodynamically significant patent ductus arteriosus [29]. Information on maternal demographics, pregnancy characteristics, and fetal ultrasound measurements was obtained from obstetric records. Data regarding neonatal characteristics and morbidities were also recorded. The present study was nested within a larger survey of FGR-exposed infants, which was approved by the Research Ethics Committee of the University Hospital of Patras (decision No. 234/2009), and written parental consent was obtained for all participants before enrollment.

### 2.2. Transthoracic Echocardiography

Cardiac anatomy and function were assessed on the second and fifth postnatal day (DOL2 at 24–36 h and DOL5 at 96–120 h, respectively) by the same pediatric cardiologist with expertise in neonatal echocardiography (AAK), using a Vivid S6 Ultrasound System (GE HealthCare, Wauwatosa, WI, USA). The infants were examined unsedated, during quiet natural sleep after regular feeding. Conventional neonatal transthoracic echocardiography was performed according to recently published standards [30].

### 2.3. Aortic Diameter and Intima–Media Thickness

Aortic diameter and intima–media thickness were measured on DOL2 and DOL5 by the same experienced investigator (AAK), using a Vivid S6 Ultrasound System (GE HealthCare, Wauwatosa, WI, USA). The aIMT was assessed in a straight, nonbranched, 1-cm-long, longitudinal segment of the distal abdominal aorta [17] and was measured as the distance between the media–adventitia and blood–intima interfaces. The aortic diameter (AoD) was calculated measuring the distance between the media–adventitia interface of the near and far wall [17,18]. Images were obtained in triplicate (i.e., 3 separate recordings) at end-diastole, coinciding with the electrocardiographic R-wave [17,18]. Images were stored digitally for subsequent offline analysis by a second investigator (SF), who was unaware of the newborns’ clinical status and ImageJ (1.54.m, 6 December 2024 01:55) was used for off-line measurements [31]. The aIMT and AoD were calculated as the average of 9 measurements (3 measurements per recording) after excluding the minimum and maximum aIMT values. aIMT values were normalized for the diameter of the vessel using the aIMT/AoD ratio. According to a previous study from our group in 14 infants, the coefficient of variation for intra-observer variability was calculated to be <3% [32].

### 2.4. OPG Levels

Quantitative determination of OPG concentrations was performed in serum samples on DOL2 and DOL5 using an enzyme immunoassay (Biomedica Medizinprodukte GmbH & Co. KG, Vienna, Austria). The blood samples were left to clot for 30 min, and the clotted samples were centrifuged at 2000× *g* for 10 min. The serum was stored at −70 °C until assayed. All measurements were performed in duplicate. Intra- and inter-assay coefficients of variation for OPG were 3.8% and 7.2%, respectively. The reported sensitivity of the assay was 0.07 pmol/L.

### 2.5. Statistics

According to previous studies, a sample size of at least 56 infants (28 per group) was considered necessary to detect a 0.2 mm difference in the mean aIMT between cases and controls, with 95% power at the 0.05 level. Sample size estimation was performed using G*Power software (3.1.9.7; Heinrich-Heine-Universität Düsseldorf, Düsseldorf, Germany).

Categorical variables were compared using the chi-square test, whereas continuous variables (after testing for normality using the Kolmogorov–Smirnov test) were compared using Student’s *t*-test. Paired *t*-tests were applied to perform OPG comparisons between DOL2 and DOL5. The relationship between OPG and aIMT/AoD was assessed using Pearson’s correlation, while the relationship between aIMT/AoD and various predictors was evaluated by linear regression. IBM SPSS software version 27.0 (2020, IBM Corp., Armonk, NY, USA) was used for all analyses.

## 3. Results

The characteristics of the study population are presented in Table 1. As expected, the weight, length, body surface area, and ponderal index of the FGR infants were lower compared to controls. Cesarean section was more prevalent in the FGR group.

The AoD was comparable between the two groups, but the FGR infants had larger AoD relative to their body weight (Table 2). The FGR infants had higher aIMT (absolute value or adjusted for body weight or AoD) at both DOL2 and DOL5 (Table 2).

**Figure 1:**
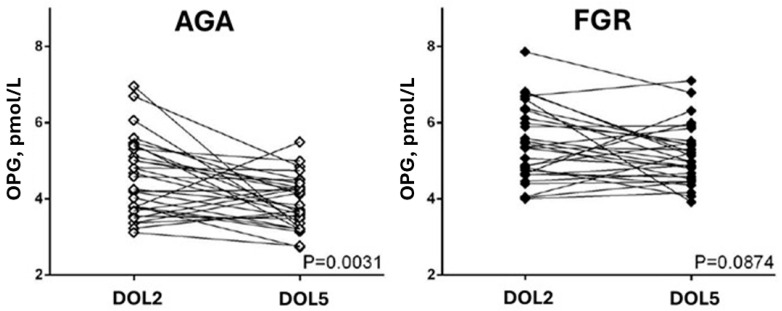
**Osteoprotegerin** (OPG) trends from DOL2 to DOL5 in the study groups. Statistical significance was assessed using paired *t*-tests. DOL: day of life.

Osteoprotegerin levels on DOL2 and DOL5 were significantly higher in infants with FGR compared to controls (DOL2: 5.4 ± 1.0 pmol/L vs. 4.6 ± 1.0 pmol/L, *p* = 0.002 and DOL5: 5.1 ± 0.8 pmol/L vs. 3.9 ± 0.7 pmol/L, *p* < 0.001). OPG levels decreased from DOL2 to DOL5 in all infants; however, this decrease was significant only in the AGA group (difference 0.7 ± 1.1 pmol/L, *p* = 0.003) and not in the infants with FGR (difference 0.3 ± 0.2 pmol/L, *p* = 0.087) (Figure 1).

**Figure 2:**
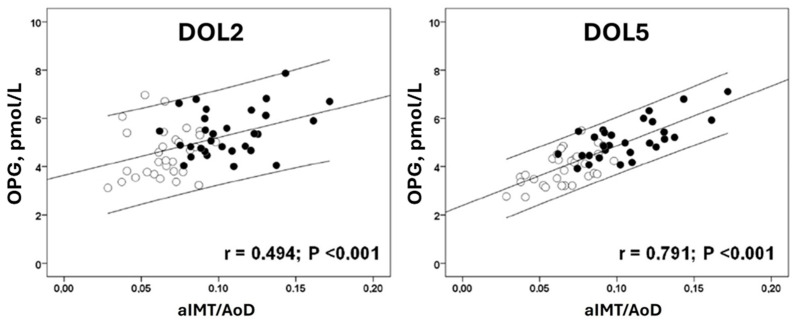
Correlation between OPG and aortic intima-media thickness/Aortic lumen diameter (aIMT/AoD) on DOL2 and DOL5. Appropriate for gestational age (AGA) (open circles) and FGR infants (closed circles) were pooled together. Pearson’s correlation line with 95% confidence intervals is also shown. DOL: day of life.

There was a positive correlation between OPG and aIMT/AοD (i.e., the aIMT adjusted for the aortic lumen diameter) on both days of measurement (DOL2: r = 0.494, *p* < 0.001; DOL5: r = 0.791, *p* < 0.001). The correlation between OPG and aIMT/AoD was stronger on DOL5 (Figure 2). OPG and FGR were the only significant independent predictors of aIMT/AoD on both DOL2 and DOL5, as shown by linear regression analysis (Table 3).

## 4. Discussion

Cardiovascular disease caused by atherosclerosis is the predominant cause of death in the developed world. Early atherosclerotic lesions, such as fatty streaks or lesions in the intima of coronary and aortic walls, begin in utero, and are a potentially reversible process [33].

Chronic intrauterine hypoxia is known to result in redistribution of fetal cardiac output towards the vital organs, leading to a regional expansion of the circulating blood volume [34]. Thus, the developing vascular tree is exposed to abnormal pressure and shear forces, which eventually lead to remodelling of the large, load-bearing arteries [34]. Alternatively, it has been proposed that arterial wall thickening may represent a maladaptive response to chronic substrate deprivation, mediated by modulation of specific genes at the arterial wall level [35]. Whichever the case, the remodelling process involves alterations in matrix protein synthesis and smooth muscle cell proliferation and apoptosis, mechanisms that are also involved in early stages of atherogenesis [13]. Experimental and clinical studies have demonstrated that fetal cardiac and arterial remodelling and impaired endothelial function persist into childhood and adolescence [7].

In this case-control study, we attempted to assess the possibility of preclinical atherosclerosis in infants with FGR by measuring OPG, a biochemical marker of endothelial dysfunction, and aIMT, an ultrasonographic vascular marker of endothelial dysfunction. Our study is the first to explore the potential relationship between aortic remodelling and plasma OPG concentrations in newborn infants. In the FGR group, plasma OPG was higher and decreased at a lower rate through the fifth postnatal day than in controls. Overall, the relationship between aIMT and OPG was significant and independent of other perinatal factors. This positive correlation was stronger on the fifth postnatal day, presumably indicating that endothelial dysfunction may become established over time. Although the physiological significance of OPG in the neonatal period remains unknown, our findings may suggest a link between in utero vascular remodelling and postnatal plasma OPG levels.

There is a significant amount of research data supporting that beyond its role in bone metabolism regulation through the OPG/RANKL/RANK axis, OPG may also be involved in cardiovascular disease processes or be a prognostic indicator of their future course [36]. The exact mechanisms through which OPG may contribute to the atherosclerotic process have not been fully elucidated. One hypothesis is that OPG enhances vascular wall inflammation and the adhesion of leucocytes to the vascular endothelium. These changes, observed in the early stages of the development of endothelial dysfunction, trigger the development of atherosclerotic vascular damage [36]. It has also been proposed that OPG contributes to atherosclerosis and cardiovascular disease by inhibiting protective pathways, including the nitric oxide synthase pathway, through the inhibition of RANKL.

Data on plasma OPG in newborn infants are sparse. In a recent study, we showed that plasma OPG levels after birth are similar to those found in older infants and children, and that prenatal corticosteroid administration may be associated with increased OPG concentrations in preterm infants [37]. We have also shown that plasma OPG levels were higher at birth in infants of mothers with preeclampsia compared to controls, and that OPG was an independent predictor of increased blood pressure in the offspring [38].

With regard to aIMT, it has been excessively used in pediatric studies for non-invasive measurement of early atherosclerotic disease [39]. In our study, infants with FGR had higher aIMT than their non-FGR counterparts, both in absolute values and after adjustment for body weight. This finding is in line with previous studies reporting that FGR is associated with aortic wall thickening and enlargement of the vessel [40]. It should be noted, though, that an increased aIMT during the neonatal period may not always reflect the beginning of a pathological process. It may also represent a transient and physiological structural adaptation to decreased aortic blood flow caused by the termination of the umbilical and placental circulations [41,42].

Given that FGR is a major global health concern with a strong influence on cardiovascular health, the findings of the present study may be of clinical significance, as they may provide useful antenatal measures to identify individuals at increased risk of complications. Obstetricians, neonatologists, and pediatricians have an important “window of opportunity” to prevent and improve adult health by implementing preventive strategies, starting from fetal or early postnatal life [5,7]. The timing of placental insufficiency, the gestational age, and the severity of growth retardation are the most predictive factors for future complications [7,33]. Therefore, early recognition and timely management is crucial and may be achieved with earlier or more frequent echographic fetal growth assessment; adaptation of maternal lifestyle, including dietary modifications to ensure normal maternal weight maintenance; euglycemia and normal maternal blood pressure; lifestyle measures (e.g., avoidance of alcohol and smoking); and control of chronic diseases. The use of antenatal steroids to prevent preterm birth is also of significance [6]. Identifying the optimal timing of delivery in FGR is another critical aspect. Postnatal interventions are crucial for promoting long-term health in individuals with former FGR, including breastfeeding, early and regulated postnatal growth with adequate nutrition, and avoidance of overfeeding. Changes in maternal and offspring microbiota with prebiotics may also be beneficial [5]. A better understanding of neonatal morbidities associated with FGR using non-invasive imaging, such as aIMT, and novel potential cardiovascular biomarkers, such as the OPG–RANKL axis, may help early identification of potential adverse outcomes, thus timely provision of healthcare. Therefore, our findings may have implications for the prevention of future cardiovascular morbidity.

Our study has some limitations. Firstly, the sample size is small, which limits the statistical power and generalizability of the findings. Second, data collection for this study was performed in 2010 and 2011; however, this does not affect the contemporary relevance of our findings, as all measurements and definitions (e.g., FGR) were clearly established and aligned with existing guidelines. Third, the FGR infants studied had limited neonatal morbidity, so no safe conclusions can be drawn regarding vascular remodelling and its relation to OPG in more severe FGRs. Another limitation of the study is that measurements were limited to the first five days of life, preventing conclusions about the persistence of vascular or biochemical alterations. The studied FGR infants were also exposed to antenatal corticosteroids at a larger percentage compared to controls, which, based on our previous finding that antenatal corticosteroid exposure may result in increased serum OPG levels in offspring, may partly account for their increased OPG levels. Similarly, a positive history of confounding maternal factors, such as smoking during pregnancy, hypertension or gestational diabetes, was more often present in the FGR infants, hypothetically contributing to the elevated OPG levels and aIMT. Moreover, the correlation between aIMT and OPG does not establish a causal relationship between them. In addition, the increased aIMT in FGR infants may indicate precocious vascular dysfunction or a transient effect reflecting delayed adaptation of the offspring to the postnatal environment. Therefore, larger, longitudinal studies with repeated OPG and aIMT assessments are needed to confirm the exploratory nature of our findings and determine whether increased aIMT in FGR infants is a permanent finding or a transient adaptive process, and whether its correlation with osteoprotegerin is an index of subclinical atherosclerosis in the offspring. The pathophysiological basis of vascular damage in FGR remains unknown and is likely more complex than a relationship between vessel wall thickness and OPG levels.

## 5. Conclusions

In conclusion, our study presents preliminary evidence linking fetal growth retardation to early vascular changes and altered osteoprotegerin levels in neonates. The development of validated non-invasive tools for identifying early atherosclerotic processes has the potential to identify vascular dysfunction or aberrant vascular growth long before vascular damage becomes clinically apparent, thereby enabling early intervention. Measuring aortic intimal thickening morphometrically is necessary to assess the significance of these relationships in the neonatal period. Additional follow-up studies are needed to determine whether the present findings represent a transient or permanent consequence of FGR. Additionally, the mechanisms underlying the correlation between aIMT and OPG remain unclear; however, involvement of the OPG/RANKL axis in cardiovascular adaptation may be suggested. The above associations should be a focus of future longitudinal research to assess subsequent cardiovascular risk among infants or children exposed to FGR.

## Figures and Tables

**Table 1:** Characteristics of the study population.

	AGA	FGR	*p* *
n	30	30	
Male sex, *n* (%)	14 (46.7)	12 (40.0)	0.793
Gestational age, weeks	36.2 ± 2.4	36.1 ± 2.7	0.880
Prematurity, *n* (%)	23 (76.7)	23 (76.7)	0.760
Cesarean section, *n* (%)	16 (53.3)	26 (86.7)	0.011
Birth weight, g	2670 ± 130	1670 ± 70	<0.001
Birth length, cm	47.7 ± 4.1	42.1 ± 3.7	<0.001
Ponderal index, kg/m^3^	25.2 ± 2.5	21.5 ± 2.9	<0.001
Body surface area, m^2^	0.19 ± 0.02	0.14 ± 0.02	<0.001
Maternal age, years	31.2 ± 4.9	31.0 ± 4.0	0.863
Maternal BMI, kg/m^2^	24.6 ± 2.0	24.0 ± 2.1	0.262
Smoking in pregnancy, *n* (%)	3 (10.0)	10 (33.3)	0.060
Gestational diabetes, *n* (%)	1 (3.3)	3 (10.0)	0.601
Hypertensive disorders of pregnancy, *n* (%)	2 (6.7)	6 (20)	0.256
Antenatal corticosteroids, *n* (%)	9 (30.0)	16 (53.3)	0.117

* Chi-square test or Student’s *t*-test, as appropriate. AGA: appropriate for gestational age, FGR: fetal growth retardation, BMI: body mass index.

**Table 2:** Aortic ultrasonography.

	AGA (n = 30)	FGR (n = 30)	*p* *
DOL2			
aIMT, mm	0.33 ± 0.03	0.51 ± 0.04	<0.001
AoD, mm	5.48 ± 0.25	5.70 ± 0.21	0.323
aIMT per body weight, mm/kg	0.14 ± 0.02	0.27 ± 0.02	<0.001
AoD per body weight, mm/kg	1.95 ± 0.09	3.60 ± 0.11	<0.001
aIMT per body surface area, mm/m^2^	1.75 ± 0.14	3.45 ± 0.09	<0.001
AoD per body surface area, mm/m^2^	28.9 ± 1.31	39.9 ± 2.45	<0.001
aIMT/AoD	0.062 ± 0.004	0.94 ± 0.007	<0.001
DOL5			
aIMT, mm	0.35 ± 0.02	0.49 ± 0.03	<0.001
AoD, mm	5.42 ± 0.22	5.71 ± 0.19	0.204
aIMT per body weight, mm/kg	0.14 ± 0.01	0.26 ± 0.02	<0.001
AoD per body weight, mm/kg	1.92 ± 0.08	3.63 ± 0.10	<0.001
aIMT per body surface area, mm/m^2^	1.76 ± 0.13	3.4 ± 0.11	<0.001
AoD per body surface area, mm/m^2^	28.4 ± 1.07	40.2 ± 2.51	<0.001
aIMT/AoD	0.065 ± 0.003	0.106 ± 0.005	<0.001

* Student’s *t*-test. AGA: appropriate for gestational age, FGR: fetal growth retardation, DOL: day of life, aIMT: aortic intima-media thickness, AoD: aortic lumen diameter.

**Table 3:** aIMT/AoD predictors.

Predictors	DOL2	*p*	DOL5	*p*
	(R^2^ = 0.50)		(R^2^ = 0.66)	
OPG	0.244	0.035	0.593	<0.001
FGR	0.568	<0.001	0.279	0.017
Maternal age	0.003	0.961	0.011	0.868
Maternal BMI	−0.076	0.513	0.004	0.969
Gestational diabetes	−0.078	0.436	−0.038	0.643
Hypertensive disorders of pregnancy	−0.063	0.557	0.031	0.726
Smoking in pregnancy	0.159	0.155	0.082	0.349
Antenatal corticosteroids	−0.002	0.988	0.008	0.933

Linear regression analysis using aIMT/AoD as the dependent variable. The adjusted R^2^ for each model is presented. aIMT: aortic intima–media thickness, AoD: aortic lumen diameter, DOL: day of life, FGR: fetal growth retardation, BMI: body mass index.

## Data Availability

The data that support the findings of this study are available from the corresponding author upon reasonable request.

## Abbreviations

The following abbreviations are used in this manuscript:

FGR	Fetal growth retardation
OPG	Osteoprotegerin
aIMT	Aortic intima–media thickness
AGA	Appropriate for gestational age
DOL2	Second day of life
DOL5	Fifth day of life
DOHaD	Developmental origins of health and disease
cIMT	Carotid intima media thickness
AoD	Aortic diameter
